# Identification of Novel Somatic *TP53* Mutations in Patients with High-Grade Serous Ovarian Cancer (HGSOC) Using Next-Generation Sequencing (NGS)

**DOI:** 10.3390/ijms19051510

**Published:** 2018-05-18

**Authors:** Marica Garziera, Erika Cecchin, Vincenzo Canzonieri, Roberto Sorio, Giorgio Giorda, Simona Scalone, Elena De Mattia, Rossana Roncato, Sara Gagno, Elena Poletto, Loredana Romanato, Franca Sartor, Jerry Polesel, Giuseppe Toffoli

**Affiliations:** 1 Experimental and Clinical Pharmacology Unit, CRO Aviano-National Cancer Institute, IRCCS, via F. Gallini 2, 33081 Aviano (PN), Italy; ececchin@cro.it (E.C.); edemattia@cro.it (E.D.M.); rroncato@cro.it (R.R.); sgagno@cro.it (S.G.); lromanato@cro.it (L.R.); fsartor@cro.it (F.S.); gtoffoli@cro.it (G.T.); 2 Pathology Unit, CRO Aviano-National Cancer Institute, IRCCS, via F. Gallini 2, 33081 Aviano (PN), Italy; vcanzonieri@cro.it; 3 Medical Oncology Unit C, CRO Aviano-National Cancer Institute, IRCCS, via F. Gallini 2, 33081 Aviano (PN), Italy; rsorio@cro.it (R.S.); sscalone@cro.it (S.S.); 4 Gynecological Oncology Unit, CRO Aviano-National Cancer Institute, IRCCS, via F. Gallini 2, 33081 Aviano (PN), Italy; ggiorda@cro.it; 5 Medical Oncology Department, Azienda Sanitaria Universitaria Integrata di Udine, via Pozzuolo 330, 33100 Udine (UD), Italy; polettoelena@libero.it; 6 Unit of Cancer Epidemiology, CRO Aviano-National Cancer Institute, IRCCS, via F. Gallini 2, 33081 Aviano (PN), Italy; polesel@cro.it

**Keywords:** *TP53* gene mutations, high-grade serous ovarian cancer (HGSOC), next-generation sequencing (NGS)

## Abstract

Somatic mutations in *TP53* are a hallmark of high-grade serous ovarian cancer (HGSOC), although their prognostic and predictive value as markers is not well defined. Next-generation sequencing (NGS) can identify novel mutations with high sensitivity, that may be repurposed as potential druggable anti-cancer targets and aid in therapeutic decisions. Here, a commercial NGS cancer panel comprising 26 genes, including *TP53*, was used to identify new genetic markers of platinum resistance and patient prognosis in a retrospective set of patients diagnosed with epithelial ovarian cancer. Six novel *TP53* somatic mutations in untreated tumors from six distinct patients diagnosed with HGSOC were identified: *TP53* c.728_739delTGGGCGGCATGA (p.Met243_Met247del, in-frame insertion or deletion (INDEL); *TP53* c.795_809delGGGACGGAACAGCTT (p.Gly266_Phe270del, in-frame INDEL); *TP53* c.826_827GC>AT (p.Ala276Ile, missense); *TP53* c.1022insT (p.Arg342Profs*5, frameshift INDEL); *TP53* c.1180delT (p.Ter394Aspfs*28, frameshift INDEL); and *TP53* c.573insT (p.Gln192Serfs*17, frameshift INDEL). Novel *TP53* variants were validated by classical sequencing methods and their impact on protein expression in tumors explored by immunohistochemistry. Further insights into the potential functional effect of the mutations were obtained by different in silico approaches, bioinformatics tools, and structural modeling. This discovery of previously unreported *TP53* somatic mutations provides an opportunity to translate NGS technology into personalized medicine and identify new potential targets for therapeutic applications.

## 1. Introduction

Epithelial ovarian cancer is the leading cause of death from gynecological malignancies, with most patients (~75%) diagnosed in advanced stages, mainly FIGO (Fédération Internationale de Ginécologie et d’Obstetrique) stage III, IV [1]. Despite the initial response to surgical debulking and first-line (I-line) chemotherapy with carboplatin-paclitaxel, ~70% of patients will relapse in the first three years with the development of drug resistance [2]. The most important clinicopathological factors related to patient outcome are tumor stage and residual disease after initial surgery [3], though the ovarian histology has demonstrated a different prognostic value [4]. Two main pathways of epithelial ovarian carcinogenesis are recognized, representing two distinct main groups based on pathological and molecular characteristics: type I and type II ovarian tumors [5]. Type I carcinomas represent only 5–10% of all epithelial ovarian tumors, are typically *KRAS*, *PIK3CA*, *PTEN*, or *BRAF* mutated, and diagnosed frequently at early stages. Type I includes low-grade serous, mucinous, clear cell, and endometroid ovarian carcinomas [6]. Type II tumors are frequently *TP53* mutated and account for 90% of deaths from ovarian cancer due to late diagnosis in the advanced stage. Type II includes undifferentiated carcinomas, carcinosarcomas, and the most prevalent type of ovarian cancer predictive of a poor prognosis, high-grade serous ovarian cancer (HGSOC) [7].

A hallmark of type II HGSOC tumors is a high frequency (close to 100%) of mutations in *TP53*, whereas low-grade type I serous ovarian cancers are rarely mutated in this gene [8,9,10]. Furthermore, about 20% of HGSOCs are also mutated in the susceptibility *BRCA1/2* genes, due to a combination of germline and somatic mutations; inheritable mutations in *BRCA* genes, especially in *BRCA1*, increase the risk to develop ovarian and breast cancers [2]. The p53 tumor suppressor is a key transcription factor regulating genes involved in DNA repair, apoptosis, senescence, and metabolism in response to stress [11]. Mutant p53 (mutp53) results in the inactivation of p53 pathways and may contribute to a wide range of oncogenic pathways, making this protein highly attractive as a therapeutic target [12,13]. Mutations in p53 have been categorized as gain-of-function (GOF; or oncomorphic) [14], loss-of-function (LOF), and unclassified (Uncl) [15]. GOF mutations are characterized by the acquisition of new activities that contribute to malignant progression and resistance to anticancer treatments. A prerequisite of GOF (missense) mutations is the hyper-stabilization and accumulation of nuclear mutp53 [11,14]. To determine whether a certain *TP53* mutation enables the acquisition of new oncogenic activities and could be classified as GOF, an oncogenic phenotype should emerge from in vitro (cultured cells) and/or in vivo (animal models) study [11,16]. The main effect of LOF (nonsense, frameshift insertion or deletion (INDEL), that cause significant disruption in the translated protein) mutations is to deprive the cells of anticancer protection; they are associated with a loss of p53 protein expression. Moreover, a mutp53 isoform can exert dominant-negative (DN) effects in which the mutant allele masks the function of the wild-type (wt) allele; if the wt allele is retained, the cell may be rendered devoid of wtp53 function through such a mechanism, particularly if the mutant protein is expressed in excess over its wt counterpart [8,17,18]. Correlations between patient survival or the development of chemoresistance and the presence of a GOF/LOF/Uncl *TP53* mutation have been attempted to be found in many different clinical studies of ovarian cancer, but the results have been conflicting, probably due to an uncorrected categorization or undefined oncogenic phenotype of mutp53 [19].

In the last few years, next-generation sequencing (NGS) technology has been widely implemented and applied to characterize the genetic profile of patients due to its improved sensitivity in gene mutation detection, fast turnaround time, and reduced costs compared to traditional sequencing methods [20,21]. One of the main clinical applications of NGS is in the molecular characterization of tumors to guide therapeutic decision-making and to identify new druggable targets at both the germinal and somatic level [22]. NGS also allows the simultaneous sequencing of multiple genes (gene panels) in a single assay [23] and can be applied with minimal invasiveness—i.e., liquid biopsy—to help in early cancer diagnosis and monitoring of disease progression, drug response, and resistance [24,25].

Here, we report six novel *TP53* somatic mutations in chemo-naïve tumor tissues from six distinct patients with HGSOC using a commercial NGS gene panel. The de novo NGS variants in *TP53* were validated and interpreted for potential functional impacts at the p53 protein level using immunostaining and different in silico approaches.

## 2. Results

### 2.1. Patient Characteristics

The main clinical and demographic characteristics of the patients carrying one of the novel *TP53* somatic mutations identified here are summarized in Table 1.

### 2.2. Identification of Novel TP53 Somatic Mutations by NGS

NGS-based analysis of *TP53* was performed using Variant Studio after filtering genetic variants per the following criteria: passing (PASS) filter, variant call quality = 100, frequency of the alternative (Alt) allele ≥ 4%, and the total number of reads passing quality filters (read depth) ≥ 1000×. Six distinct de novo somatic mutations in heterozygosis affecting the coding region of the *TP53* gene were identified in six distinct patients with HGSOC. One mutation was a nonsynonymous (missense) variant and five mutations were INDEL alterations (Table 2).

The identified variants were automatically annotated against the human *TP53* reference genomic sequence NC_000017.10 (chr 17:7,571,720–7,590,868) corresponding to isoform NM_000546.5. The latest release of the International Agency for Research on Cancer (IARC) *TP53* Mutation Database (Database R18, released on April 2016), “IARC TP53 Database” (http://p53.iarc.fr/TP53GeneVariations.aspx), was used to check the de novo *TP53* variants, confirming our novel findings.

**Figure 1:**
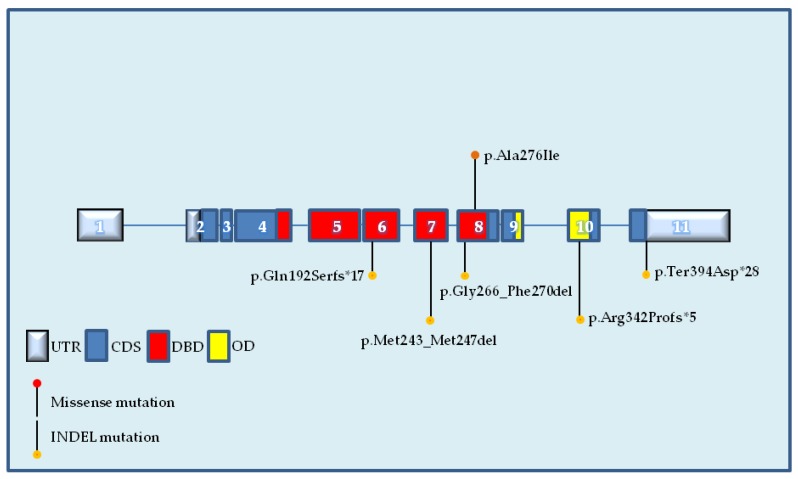
Distribution of novel somatic mutations detected by NGS along the human *TP53* gene in HGSOC. UTR: untranslated region; CDS: coding sequence; DBD: DNA binding domain; OD: oligomerization domain; INDEL: insertion/deletion; Ala: alanine; Ile: isoleucine; Arg: arginine; Pro: proline; Ter: termination; Asp: aspartic acid; Gln: glutamine; Ser: serine; fs: frameshift.

In Case ID 305, the c.728_739delTGGGCGGCATGA deletion of 12 nucleotides in exon 7 of *TP53* was identified with an allele frequency of 58,4% (Table 2). This is an in-frame INDEL mutation that causes the deletion of the second and third nucleotide in codon 243 (ATG; deleted nucleotides are underlined) encoding a methionine and the 12 subsequent nucleotides, including the first nucleotide in codon 247 (AAC) encoding asparagine 247 (p53 p.Met243_Met247del). Thus, the protein coding frame is preserved, with the translation of an asparagine residue (Asn) from the nucleotides (AAC) in codons 243 and 247, but with the removal of four amino acid (AA) residues (Met243, Gly244, Gly245, Met246) in the mature p53 protein at the DNA binding domain (DBD) (Figure 1).

In Case ID 519, the c.795_809delGGGACGGAACAGCTT deletion of 15 nucleotides in exon 8 of *TP53* was identified with an allele frequency of 47.8% (Table 2). This is another in-frame INDEL mutation that causes the removal of five AAs, including glycine 266 to phenylalanine 270 (p53 p.Gly266_Phe270del) at the DBD (Figure 1).

In Case ID 627, the c.826_827GC>AT tandem substitution of two nucleotides in exon 8 of *TP53* was identified with an allele frequency of 22.3% (Table 2). The consequence of this alteration is a missense mutation, with an AA change from alanine to isoleucine in codon 276 (p53 p.Ala276Ile) at the DBD (Figure 1).

In Case ID 738, the c.1022insT insertion of a thymine nucleotide in exon 10 of *TP53* was identified with an allele frequency of 53.4% (Table 2). This alteration produces a frameshift INDEL mutation with an AA change from arginine to proline at codon 342 (p53 p.Arg342Profs*5) and the creation of a premature stop signal after five codons. This novel variant is localized in the oligomerization domain (OD) at the C′-terminal region of p53 (Figure 1).

In Case ID 751, the c.1180delT deletion of a thymine nucleotide in exon 11 of *TP53* with an allele frequency of 8.3% was identified. This deletion produces a frameshift INDEL mutation that localizes in the final stop codon of p53 protein (codon 394), leading to an AA change to aspartic acid (p53 p.Ter394Aspfs*28) and the creation of a stop signal after 28 residues.

In Case ID 761, the c.573insT insertion of a thymine nucleotide in exon 6 of *TP53* was identified with an allele frequency of 73.3% (Table 2). This genetic variation leads to another frameshift INDEL mutation that leads to an AA change from glutamine to serine at codon 192 (p53 p.Gln192Serfs*17) and the creation of a precocious stop signal after 17 residues at the DBD.

Each ovarian cancer sample presented in this report was found to be mutated only in *TP53*; mutations were not detected in the 25 other cancer genes included in the NGS tumor panel (*BRCA1/2* genes are not included in the panel).

### 2.3. Validation of De Novo TP53 Somatic Mutations in HGSOC

**Figure 2:**
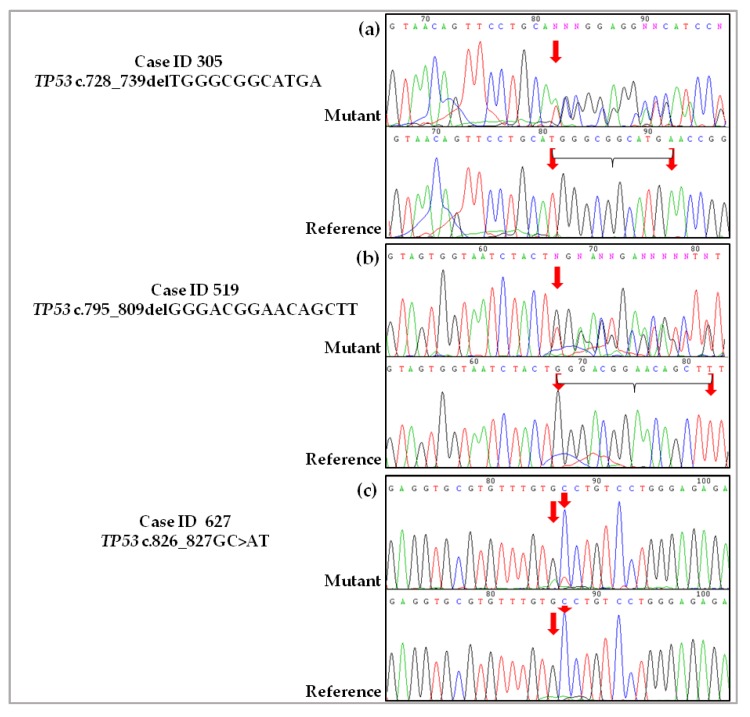

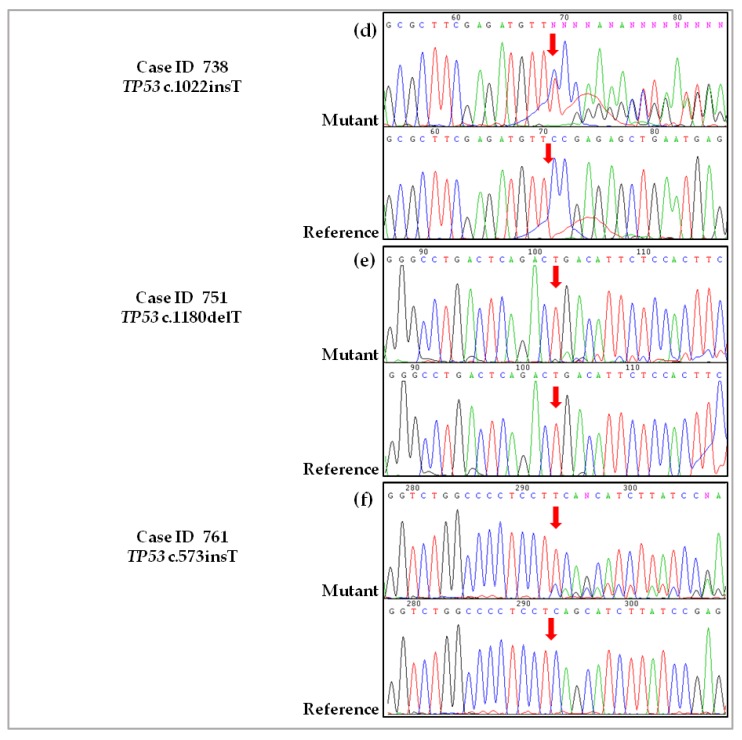
Sanger sequencing validation of novel *TP53* somatic mutations discovered in HGSOC by NGS. The chromatograms show the sequences of novel *TP53* mutations relative to genomic DNA from tumor tissue samples (Mutant) and the wt sequence related to genomic DNA isolated from PBMCs (Reference) from the matched patient. The position of nucleotide substitution is indicated by a red arrow. (**a**) Case ID 305, novel heterozygous somatic mutation in exon 7 (in-frame INDEL). The 12 deleted nucleotides are highlighted in the reference chromatogram; (**b**) Case ID 519, novel heterozygous somatic mutation in exon 8 (in-frame INDEL). The 15 deleted nucleotides deleted are highlighted in the Reference chromatogram; (**c**) Case ID 627, novel heterozygous somatic mutation in exon 7 (missense mutation); (**d**) Case ID 738, novel heterozygous somatic mutation in exon 10 (frameshift INDEL); (**e**) Case ID 751, novel heterozygous somatic mutation in exon 11 (frameshift INDEL); (**f**) Case ID 761, novel heterozygous somatic mutation in exon 6 (frameshift INDEL). del: deletion; ins: insertion.

The six de novo *TP53* somatic mutations identified in six patients with HGSOC by NGS were also evaluated by Sanger sequencing using the identical genomic DNA isolated from the tumor samples. As a wt reference sample, genomic DNA was isolated from matched peripheral blood mononuclear cells (PBMCs) from the same patient. In addition to the Sanger approach, pyrosequencing was performed to validate the novel *TP53* variants detected with low allele frequency (ID 751) and the carrier of a particular double tandem nucleotide change (ID 627). Sanger sequencing confirmed the novel *TP53* alterations detected by NGS in the tumor samples, as well the presence of the corresponding wt sequence in all of the matched peripheral blood samples (Figure 2).

Only in one sample (Case ID 751) was Sanger sequencing unable to clearly confirm the presence of the novel variant, as the chromatograms presented very small variant peaks (Figure 2e). The samples also underwent pyrosequencing, and the presence of the deletion was confirmed (Figure S1). In Case ID 751, another *TP53* mutation that has already been described was detected with an allele frequency of 8,9%; this known mutation, a single nucleotide change in exon 5 (*TP53* c.454C>A), leads to a missense change (p.Pro152Thr) in the DBD and was confirmed by Sanger sequencing. The novel *TP53* variant found in Case ID 627 (Figure 2c) was also validated pyrosequencing (Figure S2). The frequency of mutated alleles obtained from quantification of the peaks was confirmed to be similar to the allele frequency detected by NGS (Figures S1 and S2).

### 2.4. Immunohistochemical Evaluation of p53 Expression in Tumor Samples

**Figure 3:**
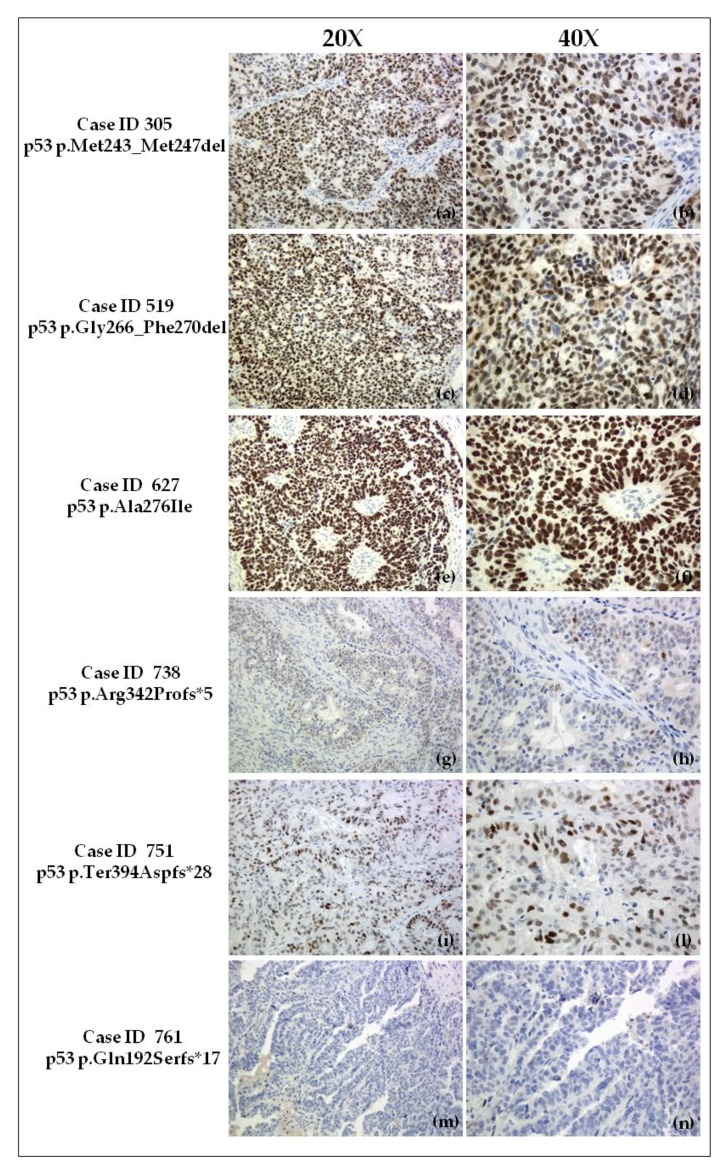
p53 immunohistochemistry of HGSOC samples carrying a novel *TP53* somatic mutation (20× and 40× magnification). (**a**,**b**) Case ID 305 (in-frame INDEL mutation), overexpression of nuclear p53 (~80% of positive tumor cells) with high intensity (++); (**c**,**d**) Case ID 519 (in-frame INDEL mutation): overexpression of nuclear p53 (~80% positive tumor cells) with high intensity (++); (**e**,**f**) Case ID 627 (missense mutation), strong overexpression of nuclear p53 (~100% positive tumor cells) with very high intensity (+++); (**g**,**h**) Case ID 738 (frameshift INDEL mutation), underexpression of nuclear p53 equivalent to p53 (−) (<5% of tumor cells with weak staining (−/+)); (**i**,**l**) Case ID 751 (frameshift INDEL mutation), moderate nuclear p53 overexpression (~60% positive tumor cells) with ~30% with moderate (+) and ~30% high (++) intensity; (**m**,**n**) Case ID 761 (frameshift INDEL mutation), completely absent nuclear p53 expression (−). No cytoplasmic p53 staining was observed. del: deletion; Met: methionine; Gly: glycine; Phe: phenylalanine; Ala: alanine; Ile: isoleucine; Arg: arginine; Pro: proline; Ter: termination; Asp: aspartic acid; Gln: glutamine; Ser: serine; fs: frameshift.

The highest percentage (close to 100%) of p53-positive tumor cell nuclei, with very high intensity (+++), was detected in Case ID 627 (Figure 3e,f). Both Case ID 305 (Figure 3a,b) and Case ID 519 (Figure 3c,d) exhibited strong p53 nuclear overexpression with ~80% positive tumor cells with high intensity (++). Case ID 751 had approximately 60% positive tumor cells, but with a different staining intensity (globally +): ~30% with moderate (+) and ~30% with high (++) intensity (Figure 3i,l). Case ID 738 was estimated to have <5% of positive tumor cells with weak (−/+) staining, equivalent to p53-negative (Figure 3g,h). A complete absence (−) of p53 staining (Figure 3m,n) was detected in Case ID 761. Cytoplasmic staining was not observed in any of the analyzed samples. Due to loss of p53 expression, frameshift INDEL mutations in Case ID 738 and Case ID 761 may be classified as LOF, whereas the remaining variants are Uncl [15].

### 2.5. In Silico Prediction and Structural Analysis

In silico analysis of the unique missense mutation found in Case ID 627 predicted a possibly damaging effect according to PolyPhen, damaging according to SIFT, and disease-causing (i.e., probably deleterious) according to MutationTaster tool (Table S1). All of the remaining novel TP53 somatic mutations detected were prognosticated with high probability to have a disease-causing effect according to MutationTaster, with the exception of the mutation in Case ID 751, which was predicted as “polymorphism” (i.e., harmless) by this in silico tool (Table S1). The Human Splice Finder (HSF) predicted a potential alteration of splicing for all novel variants discovered except for those detected in Case ID 627 and Case ID 751 (Table S1). Multiple alignment of p53 mature protein sequences from different species using the Clustal Omega tool showed that AA consensus residues in positions in which the mutations in Case ID 627 and Case ID 305 were detected are localized in highly conserved regions (Figure S3); these variants do not cause translation of truncated proteins. The mutation in Case ID 519 exhibited a minor degree of conservation, particularly in the asparagine 268 (Asn268) and serine 269 (Ser269) residues, which were mutated in different organisms (Figure S3). The first consensus residue mutated in the frameshift INDELs found in Case ID 738 and Case ID 761 was conserved in 80% and 90% of the compared p53 sequences, respectively.

Three-dimensional (3D) structural analysis showed that the mutation in Case ID 305 is localized in the L3 loop (Met237-Pro250) (Figure S4a–d), one of two important loops forming part of the DNA minor groove binding surface [26]. Deletion of these residues (Met243, Gly244, Gly245, Met246) suggests a strong rearrangement in this key region for maintenance of wtp53 transcriptional activities. The L3 loop has been reported to be crucial for interactions between the DBD and DNA minor groove; in particular, the two methionines (Met243, Met246) connected by two flexible Gly residues (Gly244, Gly245) are referred to as the “methionine switch”, which is responsible for a large conformational transition in L3 [26]. The mutation in Case ID 627 results in a change to another hydrophobic AA (Ile) and is localized in the short loop (Cys275-Cys277) between the S10 β-sheet and H2 α-helix motifs (Figure S4e–h) near the DNA major groove binding surface formed by the L1 loop (Phe113-Thr123) and short H2 helix (Pro278-Glu287) [26]. It is plausible that an additional methyl group in the side chain of the Ile residue may significantly perturb DNA-protein interactions because a relevant role for the Ala276 residue has been reported [27,28]. The in-frame INDEL mutation in Case ID 519 is clustered in the S10 β-sheet (Leu264-Val274) in proximity of the DNA major groove binding surface [26]. The five deleted AAs (Gly266, Arg267, Asn268, Ser269, Phe270) due to the mutation in Case ID 519 share different chemical properties: Gly is a key residue for flexibility of the main chain conformation, Arg is positively charged, Asn and Ser are polar residues, and Phe is a hydrophobic residue with a benzyl side chain. Thus, due to conservation of the S10 β-sheet and the entire DBD region, we can hypothesize that deletion of these residues may change the affinity of p53 for DNA target sequences. The already annotated p53 p.Pro152Thr change (Case ID 751) is localized in an α-helix between β-sheets S3 and S4 [26] and is quite dramatic because proline (Pro) is a hydrophobic residue and threonine (Thr) a polar residue and may drive H-bond formation through its oxydrilic group, interfering with the local structure [29]. The frameshift INDEL mutation in Case ID 761 occurred in the L2 loop (Lys164-Cys176, Cys182-Leu194) necessary to form with L3 loop, the DNA minor groove binding surface [26].

Furthermore, two CpG dinucleotides targeted for methylation are recognized in both in-frame INDEL p53 mutations identified in Case ID 305 (codons 244 (GGC; nucleotides forming CpG island are underlined) and 245 (GGC)) and Case ID 519 (codon 267 (CGG)).

The most relevant findings related to the novel *TP53* mutations are summarized in Table 3.

## 3. Discussion

Mutations in *TP53* are the most frequent gene alteration in human cancers and the main molecular feature of HGSOC. Here, we report six previously unreported patient-specific *TP53* mutations identified in tumor DNA from women with chemo-naïve HGSOC by NGS. The mutations were validated by classical sequencing methods and studied at the protein level (i.e., p53 expression) in each tumor sample. Further functional considerations were extrapolated through a structural modeling approach and other in silico bioinformatics tools. In only one case the Sanger method was unable to robustly confirm the presence of the variant, probably due to the low allele frequency of the mutation (<10%) (Table 2); this lower limit of detection related to the Sanger sequencing technique is supported by other studies [30,31]. However, the use of the more sensitive pyrosequencing technique allowed an analytical value for this mutation.

The p53 tumor suppressor monomer is a 393 AA protein translated by 11 exons comprising different functional domains, the most relevant of which are the central site-specific DBD (residues 102–292), the OD (also known as tetramerization domain; residues 325–356), and a strongly basic C′-terminal domain (residues 364–393) [32]. The OD has a critical value for biological p53 functions because it permits the oligomerization of p53 protein in the native tetrameric conformation necessary for transcriptional activity [33]. *TP53* mutation spectra may be very heterogeneous with the detection of in-frame or frameshift INDEL mutations, nonsense, silent, splice, and other infrequent alterations, but missense mutations leading to an AA change in the protein coding sequence are predominant (~70%) [34]. Approximately 90% of known *TP53* variants, mostly of the missense type, cluster between exons 4 and 9 in the highly conserved DBD [32], especially at specific “hot spot” codons in ovarian cancer (i.e., 273, 220, 248, 175, in decreasing order of frequency), suggesting a selective advantage for these mutants [16].

Four out of the six novel *TP53* alterations are localized at the DBD, the functional core of p53 directly involved in its interaction with DNA. The remaining two frameshift INDEL mutations were identified in the OD, specifically at the nuclear export signal (NES, residues 340–351) sequence, and in the last codon codifying for the stop signal for p53 translation at the C′-terminal domain. The finding of novel *TP53* variants outside the DBD is relevant because, in past years, in most cases mutations were searched only in the region covered by *TP53* exons 4–9. The results for Case ID 751 highlight the high sensitivity and great scientific achievements of NGS compared to standard sequencing. Recently, NGS led to the identification of several novel mutations in different types of tumors [35], including ovarian cancer [36], that may otherwise have remained undiscovered.

We observed that ovarian tumor samples from Case ID 305, 519, and 627 were mutated for novel p53 alterations that do not modify the protein frame, producing truncated p53 isoforms and resulting in marked nuclear overexpression of p53 (Figure 2a–f; Table 3). In particular, the highest level of overexpression was detected in the present of the novel missense mutation p53 p.Ala276Ile. Similarly, pathologic p53 expression (Figure 2a–d; Table 3), was reported in tumors (Case ID 305, Case ID 519) mutated for the two in-frame INDELs discovered.

The novel p53 alterations associated with nuclear accumulation and increased half-life of mutp53 are present in the DBD stabilizing regions, which can result in a loss of normal protein-protein interactions [37]. Structural integrity and DBD folding are required for wtp53 to bind to its target genes and function as a transcription factor. These mutations are localized in consensus residues that share a strong degree of phylogenetic conservation and may have important structural consequences on DBD (Table 3). A proper DBD conformation is also required for nuclear export of p53 resulting from ubiquitination by MDM2 at the p53 C′-terminal lysine (Lys) residues and the exposure and activation of the NES included in the OD, which is characterized by a highly conserved sequence [38,39]. These mutants may induce a conformational change, leading to the masking of the C′-terminal NES and impaired mutp53 degradation as evidenced by the strong nuclear p53 accumulation (Case ID 305, 519, and 627). Moreover, deletion of some CpG sites in DBD may influence the binding affinity for p53 target sequences [40,41].

To date, in vitro and in vivo functional studies of mutp53 proteins to assess their oncogenic properties have been performed only on missense mutations, especially those described in the “hot spot” codons [11]. The p53 (missense) GOF mutations localized in the DBD have been grouped in two main categories: DNA contact mutants (i.e., R248W and R273H) associated with exclusive p53 nuclear staining, and DNA structural or conformational mutants (e.g., R175H, R282W, R249S, R248Q, P250L, E258V, R110L, R110P) that exhibit cytoplasmic p53 localization [39]. Considering the absence of cytoplasmic staining of p53 in the corresponding tumor sample (Case ID 627), the novel missense mutation reported here probably shares greater affinities with contact rather than structural mutants described [19,42]. Consequently, it is plausible that p53 p.Ala276Ile may be a GOF mutation.

The meaning of this variant remains uncertain without conclusive data from functional studies, i.e., Uncl (Table 3). It is also difficult to come to a conclusion regarding the p53 in-frame INDEL mutations (Case ID 305 and Case ID 519), even if we could exclude a LOF effect via immunohistochemistry; thus, the variants should be categorized as Uncl (Table 3). p53 binds DNA as a tetramer, and it is well accepted that inactivation of wtp53 through DN effects requires tetramerization [19,43]. The mutated allele in the new *TP53* alterations in Case ID 305 and Case ID 519 may lead to the formation of mixed heterotetramers and confer unknown DN effects on the remaining wt p53 allele.

The novel frameshift INDEL mutation in Case ID 761 leads to the creation of a premature stop codon after 17 residues and the translation of a truncated protein with abrogation of the OD and most of the DBD. Furthermore, the three nuclear localization signals (NLSs) and C′-terminal NES included in the OD, which are necessary for nuclear importation and exportation of p53, respectively, are disrupted (Table 3). In particular, the first NLS (NSLI, residues 316–324) is the most active and conserved domain among NLSs, with three consecutive Lys residues to a basic core. Structural consequences on this mutp53 are confirmed by the complete absence of p53 protein expression in the corresponding tumor sample (Figure 2m,n). We concluded that it is a LOF mutp53 (Table 3). Notably, the antibody epitope used in immunohistochemistry maps to the N-terminal domain of the p53 protein [44]; the nuclear negative staining is in accordance with disruption of the OD and NLS regions and the impossibility of gaining a tetrameric functional p53 or nuclear transfer.

The novel *TP53* frameshift INDEL mutation in Case ID 738 leads to the abrogation of part of the OD, the included NES, and two of the three NLSs (NLSII residues 370–376, NLSIII residues 380–386). We hypothesize that the formation of a functional tetramer is compromised with loss of wt p53 expression levels (LOF mutation) (Table 3). Otherwise, the conservation of the NLSI segment may permit access to the nuclear space of this truncated and probably non-functional protein. This possibility may partially explain the few positive tumor cells detected by immunohistochemistry that were representative of a pathological condition (Figure 3g,h) similar to that observed in Case ID 761. The novel *TP53* mutation detected in Case ID 751 is a particular frameshift INDEL because it produces an abnormal longer p53, but with intact structural and functional domains (no truncated p53 isoform). Curiously, this sample was also a carrier of an already described missense change (p53 p.Pro152Thr) in the DBD of p53, considered an Uncl variant with no evidence of GOF activity [15,45]. A considerable number of p53 mutations are defined as Uncl due to conflicting studies on their function (i.e., splice variants) or a lack of in vitro and/or in vivo functional studies to identify an oncogenic phenotype. Most p53 Uncl variants are missense mutations [15]. Moreover, tumors with Uncl p53 mutations have a broad range of p53 protein expression [15]. The tumor sample from Case ID 751 had heterogeneous abnormal expression of p53 intermediate to that observed in Case ID 305 and Case ID 738, with a variable staining intensity in tumor cell nuclei. Another important point to consider is that INDELs, especially frameshift INDEL mutations, may also interfere with protein translation, introducing aberrant splicing, as suggested by the in silico prediction of mutations found in Case ID 738 and Case ID 751. The frameshift INDEL variant in Case ID 751 was not predicted to have a deleterious effect and a significant impact on splicing by bioinformatics tools. Finally, we reported an Uncl definition for this novel *TP53* mutation (Table 3).

Resistance to platinum and other DNA-damaging agents used in the treatment of ovarian cancer is a consequence of reduced cell susceptibility to activation of the apoptotic response associated with the inactivation of *TP53* pathways [16]. Different approaches have been applied to address the underlying mechanisms involved in platinum resistance and outcome in patients with ovarian cancer based on the characterization and impact of the *TP53*-mutated signature [40,46]. Using stringent criteria to define GOF/oncomorphic, LOF, and Uncl *TP53* somatic mutations, an association with platinum resistance and worse survival was demonstrated for patients with GOF mutp53 in advanced serous ovarian cancer [15] and HGSOC [45]; both studies used the TCGA dataset [10]. However, a *TP53*-mutated signature, independent from the type of mutp53, was associated with an improved prognosis in ovarian carcinomas in recent publications [47,48,49,50,51]. This discrepancy regarding an opposite prognostic value may be explained by the incomplete characterization of a large number of *TP53* mutations, especially missense mutations, but also in-frame/frameshift INDELs, splice sites, and others, that are currently unclassified. The already reported (but unclassified) and novel *TP53* mutations should be integrated with functional studies in cell lines and mouse models to express the ectopic mutp53.

All of the patients with HGSOC reported here, except Case ID 738 due to a lack of follow-up data on platinum-free interval (PFI) and progression-free survival (PFS), were platinum-sensitive (PFI > 12 months). Among the platinum-sensitive patients, Case ID 761 had the shortest PFI. In HGSOC, the combined loss of the OD (responsible for tetramerization) and NES domains is critical for platinum resistance [52]; the LOF mutations (frameshift INDELs) found in Case ID 738 and Case ID 761 lead to disruption of both gene segments and to the translation of truncated mutp53 isoforms.

Concerning survival, the shortest overall survival (OS) was <1 year, but prolonged survival (≥5 years) was reported for four of the cases in this study. Death occurred in two of the six patients with HGSOC during follow-up. Although the median OS reported for epithelial ovarian cancer is <5 years, approximately 15% of patients will have prolonged survival [53]. The precise genetic profile associated with this distinct and favorable prognosis is still to be identified clearly, though an immunoreactive gene signature has been highlighted [10,54]. Nonetheless, the literature remains unclear about the association between survival and a well-defined *TP53* gene signature. The most important prognostic factor in ovarian cancer is probably the tumor residual [55]. Optimal cytoreductive surgery with no visible residual (R = 0) was performed in all patients except for Case ID 738; moreover, among patients presented in this report, Case ID 738 was the oldest one at diagnosis. This data should be taken into account because it may have had a significant impact on determining survival for this patient or gaining an addictive phenotypic value in combination with the *TP53* frameshift INDEL mutation identified in the tumor. A limiting factor for this study is represented by the unknown *BRCA1/2* mutational burden that was not tested in the patients of this report, even if they have not reported a history of breast and/or ovarian cancer among their first-degree relatives. The current recommendation in the clinical practice, is to test *BRCA* genes in all patients diagnosed with epithelial ovarian cancer, especially in the high grade tumors [56] although, at present, the role of germinal *BRCA* status does not modify first-line treatment being crucial only when patients have a platinum sensitive recurrence. *BRCA* carriers have been reported to better respond to platinum-based therapy and may candidate to poly-ADP ribose polymerase (PARP) inhibitor-based therapy [57]. Moreover, the combined impact of *BRCA1* and *TP53* mutations on survival of HGSOC was recently found to influence prognosis [47].

In conclusion, we report the discovery of six novel somatic mutations in *TP53* in six distinct patients with HGSOC using the high-throughput NGS technique. Two frameshift INDEL mutations were considered LOF, and one missense, one frameshift INDEL, and two in-frame INDELs were categorized as Uncl *TP53* mutations. The functional impact and prognostic value of these novel variants remain to be elucidated. Further studies are warranted to determine whether these mutations may be potential therapeutic targets for personalized treatment. It is clear that the type of approach used to target mutp53 will depend on the type of mutation and its functional impact. Notably, the discovery of unreported *TP53* mutations provides the possibility to translate NGS technology into personalized medicine to identify new potential targets for future therapeutic applications—i.e., pharmacological reactivation of mutant p53 [12,13]—and to improve on our knowledge of the complex genomic heterogeneity of ovarian cancer. Future directions will be to define the clinical value of *TP53* somatic mutations in a large cohort of patients with advanced epithelial ovarian cancer, particularly HGSOC.

## 4. Materials and Methods

### 4.1. Patients and Human Ethics

Written informed consent was obtained from each patient with histologically confirmed epithelial ovarian cancer for the use of peripheral blood, tissue samples, and clinical data for research purposes. The study was conducted in accordance with the Declaration of Helsinki and was approved by the Ethics Committee of the CRO Aviano National Cancer Institute, Italy (Institutional Review Board n. CRO-2014-43, (16 December 2014). Patients included in this report were diagnosed and treated at CRO Institute between August 2004 and December 2011. The novel *TP53* somatic mutations emerged during a genomics screen aimed to identify new genetic markers of platinum resistance and patient prognosis in epithelial ovarian cancer using a targeted NGS approach. Frozen ovarian tumor specimens taken during primary surgery before any chemotherapeutic treatment or during diagnostic laparoscopy and matched blood samples were analyzed retrospectively. Clinico-pathological characteristics and treatment and prognosis information were collected from medical records. OS was defined as the interval between primary debulking surgery (PDS) or interval debulking surgery (IDS) and the date of death for any cause or last follow-up. PFS was defined as the interval between PDS or IDS and the date of first recurrence/progression or last follow-up. PFI was defined as the interval between the end of the first-line platinum-based treatment and the date of first recurrence/progression or last follow-up. If relapse occurred during treatment or within four weeks of the end of platinum, the patient was defined as ‘refractory’; if the interval was <6 months, the patient was defined as ‘platinum-resistant’; if the interval was between 6 and 12 months, the patient was ‘intermediately sensitive’; if the interval was >12 months, the patient was defined as ‘platinum-sensitive’.

### 4.2. Case History

#### 4.2.1. Case ID 305

A 58-year-old female was diagnosed with grade 3 FIGO stage IIIC serous ovarian cancer after PDS with hysterectomy, bilateral salpingo-oophorectomy, omentectomy, appendectomy, and partial intestinal resection (absence of visible residual tumor). PDS was followed by six cycles of standard first-line chemotherapy with carboplatin and paclitaxel. Death occurred due to cerebral hemorrhage after 43 months without any sign of recurrence. The PFI was 38 months; therefore, the patient was defined as platinum-sensitive. PFS and OS were 43 months.

#### 4.2.2. Case ID 519

The patient was diagnosed at 52 years of age with grade 3 FIGO stage IV (extra-regional lymph node involvement) serous (a minimal component, <1% with morphological aspects of clear cells observed by the pathologist) ovarian cancer after PDS with hysterectomy, bilateral salpingo-oophorectomy, omentectomy, perinectomy, appendectomy, and systematic pelvic and aortic lymphadenectomy (absence of visible residual tumor). PDS was followed by six cycles of standard first-line chemotherapy with carboplatin and paclitaxel. Abdominal recurrence was instrumentally detected after 85 months. The patient was defined as platinum-sensitive (PFI 80 months) and the PFS was 85 months. She was still alive at the date of the last follow-up, 98 months after the diagnosis.

#### 4.2.3. Case ID 627

The patient was diagnosed at 68 years of age with epithelial ovarian cancer with unresectable tumor (residual disease >2 cm) and presence of abundant ascites (clinical stage IV) after a diagnostic laparoscopy (collection of multiple biopsies). The patient was assigned to three cycles (carboplatin and paclitaxel) of neo-adjuvant chemotherapy (NACT) followed by IDS and three additional cycles of carboplatin plus paclitaxel. During IDS, the patient was staged grade 3 FIGO stage IIA serous ovarian cancer with bilateral salpingo-oophorectomy (hysterectomy was performed two years prior), omentectomy, and systematic pelvic and aortic lymphadenectomy (absence of visible residual tumor). After 71 months, disease recurrence was clinically and instrumentally evidenced in the extra-abdominal lymph nodes at the right axillary region. Surgical removal of the relapse was performed and a cytological examination confirmed the metastatic nature of the lesion. Post-operatively, the patient received carboplatin, but after the third cycle she developed an allergic reaction and the chemotherapy was stopped. The patient was defined as platinum-sensitive (PFI 64 months) and the PFS was 71 months. She was still alive at the date of the last follow-up 77 months from the diagnosis.

#### 4.2.4. Case ID 738

A 79-year-old patient was diagnosed with grade 3 FIGO stage IIC serous ovarian cancer after PDS with hysterectomy, bilateral salpingo-oophorectomy, and infracolic omentectomy (residual tumor >1 cm at abdominal lymph nodes). After PDS, standard first-line chemotherapy with carboplatin and paclitaxel was proposed but the patient continued appropriate treatment at another institution. The patient died before the last follow-up; the OS was nine months.

#### 4.2.5. Case ID 751

The patient was diagnosed at 71 years of age with grade 3 FIGO stage IIC serous ovarian cancer after PDS with bilateral salpingo-oophorectomy, radical omentectomy, appendectomy, and sigma resection (absence of visible residual tumor). PDS was followed by six cycles of standard first-line chemotherapy with carboplatin and paclitaxel. Recurrence was instrumentally detected in the liver and at the biochemical level with increased CA-125 after 34 months. The patient was treated with carboplatin and pegylated liposomal doxorubicin (Caelyx); after the eighth cycle, the patient developed a moderate allergic reaction to carboplatinum that was avoided in the last ninth cycle. After two months, peritoneal (hepatic glissonian) progression was detected with concomitant biochemical progression. The patient started treatment with gemcitabine (Gemzar), but the chemotherapy was stopped after the sixth cycle due to disease progression (increase in peritoneal lesions and increased CA-125). The patient started fourth-line chemotherapy with weekly topotecan (Hycamtin) and, after four cycles, had increased plasma CA-125 levels (progression suspected but not confirmed). The patient was defined as platinum-sensitive (PFI 29 months) and PFS was 34 months. The patient was still alive at the date of the last follow-up 60 months from the diagnosis.

#### 4.2.6. Case ID 761

The patient was diagnosed at 56 years of age with grade 3 FIGO stage IIIC serous ovarian cancer after PDS with hysterectomy, bilateral salpingo-oophorectomy, omentectomy, perinectomy, and appendectomy (absence of visible residual tumor). PDS was followed by six cycles of standard first-line chemotherapy with carboplatin and paclitaxel. After 14 months, recurrence was instrumentally detected in the peritoneum and at the biochemical level with increased CA-125. Second-line chemotherapy was based on carboplatinum plus Caelyx but, after three cycles, the treatment was stopped due to peritoneal progression and marked increase in CA-125. Despite weekly paclitaxel administration, the patient was still in disease progression (clinical, peritoneal, and biochemical) after eight months. Gemzar was administered as fourth-line treatment but, after 10 months, the patient relapsed again (clinical, peritoneal, and biochemical documentation) with abundant abdominal ascites. The patient started carboplatin again as fifth-line chemotherapy and the last follow-up recorded after one cycle, with concomitant presence of abundant ascites. The patient was defined as platinum-sensitive (PFI 14 months) and the PFS was 20 months. The patient was still alive at the date of the last follow-up 58 months from the diagnosis.

### 4.3. Next Generation Sequencing Analysis

Frozen ovarian tumor specimens taken at primary surgery or during diagnostic laparoscopy and matched blood samples were analyzed retrospectively. Tumor samples were macrodissected and visually inspected by the pathologist to assess a minimum tumor cellularity of 70%. Genomic DNA was extracted from both the tumor and PBMCs using the EZ1 DNA Tissue Kit and EZ1 DNA Blood 350 µL Kit (Qiagen, Hilden, Germany), respectively, according to the manufacturer’s instructions. DNA samples were quantified using PicoGreen Dye (Quant-iT PicoGreen dsDNA Assay Kit, Thermo Fisher Scientific, Waltham, MA, USA) on a Tecan Infinite 200 PRO reader (Tecan Trading AG, Männedorf, Switzerland) and normalized to 5 ng/μL for successive library preparation. DNA libraries were prepared for NGS using Illumina TruSight Tumor 26 genes panel (Illumina, Inc., San Diego, CA, USA) according to the manufacturer’s instructions. This panel (21 Kb size) screens 82 exons (all 11 exons of *TP53* and intronic boundaries) in 26 tumor-related genes across 174 amplicons (165–195 bp in size) with a 1000× minimum coverage (mean 7000×) of each amplicon. Details on the manufacturer’s list of targeted genes and regions can be found at http://www.illumina.com/products/trusight-tumor-26-gene.html. Normalized libraries were analyzed on a MiSeq platform (Illumina) using a V3 (600 cycles) sequencing flow cell with a 2 × 121 base pairs analysis set-up. The raw data were automatically processed and analyzed by the Illumina-Miseq system pipeline. VCF files were imported in Variant Studio software (Illumina Variant Studio Data Analysis Software 2.2) for variant calling and imputation.

### 4.4. Sanger Sequencing and Pyrosequencing to Validate Novel Somatic Mutations

Sanger sequencing was performed to validate the six novel variants identified in *TP53* using genomic DNA extracted from tumor tissue and blood from each patient. Mutations were confirmed by at least two independent PCR amplifications and a DNA sequencing reaction on both strands when possible (bi-directional or only in forward strand in presence of INDELs). Primers used to amplify *TP53* exons (Table S2) were downloaded from the public “IARC TP53 Database” (http://p53.iarc.fr/ProtocolsandTools.aspx). Target regions were PCR amplified from extracted genomic DNA; each 50 µL reaction contained 1.8 mM MgCl_2_, 0.25 mM of each dNTP, 0.5 µM of each primer, 20–200 ng of genomic purified DNA template, 1× PCR Buffer, 0.2 µL of AmpliTaq Gold DNA polymerase (Applied Biosystems, Foster City, CA, USA), and MilliQ water. The PCR protocol was as follows: initial denaturation at 94 °C for 2 min, then 50 cycles of subsequent denaturation at 95 °C for 30 s, annealing at 57 °C (53 °C for exon region 8–9) for 45 s, and extension at 72 °C for 1 min, followed by the final extension step at 72 °C for 10 min. The PCR products (5 µL) were electrophoresed in 3% agarose and amplified samples cleaned up using ExoProStar (GE Healthcare, Little Chalfont, UK). Purified reactions (1–2 μL) were sequenced using the Big Dye Terminator kit (Applied Biosystems, Foster City, CA, USA) and an ABI PRISM capillary sequencer with both forward and reverse primers when possible. Sequencing chromatograms were visualized with Chromas software version 2.01 (Technelysium Pty Ltd., South Brisbane, Australia) and aligned with the human *TP53* reference genomic sequence.

The novel *TP53* somatic variants that were difficult to interpret (i.e., Case ID 627, double tandem change and Case ID 751, lowest alternative allele frequency) also underwent pyrosequencing with specific 5′-biotinylated primers (Table S2) designed in SNP Primer Design Software (version 1.0, Biotage AB, Upsala, Sweden). Genotyping was performed using a PSQ96 pyrosequencer (Pyrosequencing AB, Uppsala, Sweden) according to the manufacturer’s instructions.

### 4.5. p53 Immunohistochemistry

One tumor-rich sample per case, a 4-µm-thick section from a formalin-fixed, paraffin-embedded tumor tissue block, was selected for immunohistochemical analysis. Immunoperoxidase labeling was performed with the automated XT iVIEW DAB V.1 procedure on the BenchMark ULTRA IHC/ISH Staining Module, Ventana with anti-p53 (clone Bp53-11, prediluted, Ventana, Innovation Park Dr. Tucson, AZ, USA). Antigen retrieval was carried out with CC1 (Ventana). Sections were incubated with primary antibody for 16 min at 37 °C. Staining was detected using the I-View DAB detection system. All slides were reviewed by the pathologist (VC), who was blinded to molecular data. Nuclear staining was considered a positive reaction. The extent of nuclear staining was estimated to the nearest 5% level of positive tumor cells, reporting the actual percentage for each case. The intensity of staining was recorded as − (absent); −/+ (weak); + (moderate); ++ (high); or +++ (very high).

### 4.6. In Silico Analysis

To predict the potential effect of the novel somatic mutations on the structure and function of human p53 protein, MutationTaster (http://mutationtaster.org) was used. PolyPhen2 v2.2.2r398 (http://genetics.bwh.harvard.edu/pph2) and SIFT (http://sift.jcvi.org) were applied to predict the potential effect of the identified missense mutation. Clustal Omega (http://ebi.ac.uk/tools/msa/clustalo/) was used to align the reference human p53 protein (UniProtKB code P04637) with sequences from other species. The Human Splice Finder (HSF 3.0.2 released on 4 December 2017; http://umd.be/HSF3/index.html) was used to investigate the potential impact of splicing abnormalities caused by the novel somatic variants in *TP53* at non-consensus splice sites. Structural 3D analysis was performed using Swiss-PdBViewer v4.1.0 (https://spdbv.vital-it.ch/).

## Figures and Tables

**Table 1:** Main clinical and demographic characteristics of patients diagnosed with HGSOC carrying a novel *TP53* somatic mutation.

Case ID	Age ^a^	TNM	FIGO	Histology	Grade	R	CT Setting	First Line Treatment	PFI	PFS	OS	Actual Status
	(year)				(G)	(cm)			(m)	(m)	(m)	
305	58	pT3cN1M0	IIIC	Serous	G3	0	I line	Carbotaxol	38	43	43	Dead
519	52	pT3cN1M1	IV	Serous	G3	0	I line	Carbotaxol	80	85	98	Alive
627	68	ypT2aN0M0	IIA	Serous	G3	0	Neo-adj	Carbotaxol	64	71	77	Alive
738	79	pT2cNxM0	IIC	Serous	G3	>1	NA	NA	NA	NA	9	Dead
751	71	pT2cN0	IIC	Serous	G3	0	I line	Carbotaxol	29	34	60	Alive
761	56	pT3cNxM0	IIIC	Serous	G3	0	I line	Carbotaxol	14	20	58	Alive

^a^ Age at diagnosis; HGSOC: high-grade serous ovarian cancer; FIGO: Fédération Internationale de Ginécologie et d’Obstetrique; m: months; R: residual disease; CT: chemotherapy; Neo-adj: neo-adjuvant; PFI: platinum-free interval; PFS: progression-free survival; OS: overall survival; NA: data not available.

**Table 2:** Novel *TP53* somatic mutations identified by NGS in six patients with HGSOC.

Case ID	Exon	Genomic Coordinate	Alt	Ref Read Depth	Alt Read Depth	cDNA Nucleotide Change	AA Change	Mutation Type
			(%) ^a^					
305	7	17:7,577,541	58.43	15,734	9194	c.728_739delTGGGCGGCATGA	p.Met243_Met247del	In-frame
								INDEL
519	8	17:7,577,128	47.84	6377	3051	c.795_809delGGGACGGAACAGCTT	p.Gly266_Phe270del	In-frame
								INDEL
627	8	17:7,577,111	22.34	4692	1048	c.826_827GC>AT	p.Ala276Ile	Missense
738	10	17:7,574,004	53.41	4606	2460	c.1022insT	p.Arg342Profs*5	Frameshift
								INDEL
751	11	17:7,572,928	8.32	2994	249	c.1180delT	p.Ter394Aspfs*28	Frameshift
								INDEL
761	6	17:7,578,275	73.38	7205	5287	c.573insT	p.Gln192Serfs*17	Frameshift
								INDEL

^a^ Alternative allele frequency; NGS: next-generation sequencing; HGSOC: high-grade serous ovarian cancer; Ref: reference allele; Alt: alternative allele; AA: amino acid; fs: frameshift; del: deletion; ins: insertion; INDEL: insertion/deletion; Met: methionine; Gly: glycine; Phe: phenylalanine; Ala: alanine; Ile: isoleucine; Arg: arginine; Pro: proline; Ter: termination; Asp: aspartic acid; Gln: glutamine; Ser: serine.

**Table 3:** Summary of the most relevant features of the novel *TP53* somatic mutations identified by NGS in six patients with HGSOC

Case ID	Novel Mutp53	p53 Domain	p53 IHC ^a^	Predicted Damaging Effect ^b^	Phylogenetic Conservation ^c^	Predicted Splicing Effect	Structural Consequence	Loss of CpG Site ^d^	Platinum Status	OS	Classification of Novel Mutp53
305	p.Met243_Met247del	DBD	~100% (+++)	Yes	Met243 100%	Yes	Probable strong rearrangement in DNA minor groove binding surface	Yes	Sensitive	3 ≤ years < 4 ^†^	Uncl
					Gly244 100%						
					Gly245 100%						
					Met246 100%						
519	p.Gly266_Phe270del	DBD	~80% (++)	Yes	Gly266 100%	Yes	Probable change in affinity of p53 for target sequence	Yes	Sensitive	>5 years	Uncl
					Arg267 100%						
					Asn268 60%						
					Ser269 90%						
					Phe270 100%						
627	p.Ala276Ile	DBD	~80%	Yes	Ala276 100%	No	Probable perturbation in proximity of DNA major groove binding surface	No	Sensitive	>5 years	Uncl
738	p.Arg342Profs*5	OD	<5% (−/+)	Yes	Arg342 80% *	Yes	Partial loss of OD	No	NA	<1 year ^†^	LOF
							Loss of C’-terminal				
751	p.Ter394Aspfs*28	C′-terminal **	~60% (30% +; 30% ++)	No	**	No	Abnormal protein elongation	No	Sensitive	≥5 years	Uncl
761	p.Gln192Serfs*17	DBD	-	Yes	Gln192 90% *	Yes	Probable strong rearrangement in DNA minor groove binding surface	No	Sensitive	4 ≤ years < 5	LOF
							Massive loss of DBD.				
							Loss of OD and C′-terminal.				

^a^ Immunohistochemical data indicate the percentage (%) of tumor cell nuclei positive for p53 and staining intensity (+++ very high; ++ high; + moderate; −/+ weak; - absent); ^b^ summarizes the global effect from MutationTaster, SIFT, and PolyPhen bioinformatics tools; ^c^ percentage of sequences from different species with the consensus (not mutated) AA residue indicated; ^d^ at the site of the mutation; ** % is reported for only the first consensus AA residue of the frameshift INDEL mutation; * mutation in the TGA stop signal of mature p53; ^†^ patient was dead at the last follow-up. IHC: immunohistochemistry; NGS: next-generation sequencing; HGSOC: high-grade serous ovarian cancer; OS: overall survival; AA: amino acid; fs: frameshift; del: deletion; ins: insertion; INDEL: insertion/deletion; Met: methionine; Gly: glycine; Phe: phenylalanine; Ala: alanine; Ile: isoleucine; Arg: arginine; Pro: proline; Ter: termination; Asp: aspartic acid; Gln: glutamine; Ser: serine; Asn: asparagine; Nd: not determined; NA: data not available; DBD: DNA binding domain; OD: oligomerization domain; Uncl: unclassified; LOF: loss-of-function.

## Supplementary Materials

Supplementary materials can be found at http://www.mdpi.com/1422-0067/19/5/1510/s1.
