# Supplementary material for: Identification of Novel Somatic TP53 Mutations in Patients with High-Grade Serous Ovarian Cancer (HGSOC) Using Next-Generation Sequencing (NGS)
Source: Int J Mol Sci. 2018 May 18;19(5):1510. doi: 10.3390/ijms19051510 (PMC5983728; doi:10.3390/ijms19051510)
Supplement: Supplementary file 1 [file ijms-19-01510-s001.pdf]

Supplementary Material: Identification of Novel Somatic *TP53* Mutations in Patients with High-Grade Serous Ovarian Cancer Using Next-Generation Sequencing (NGS)

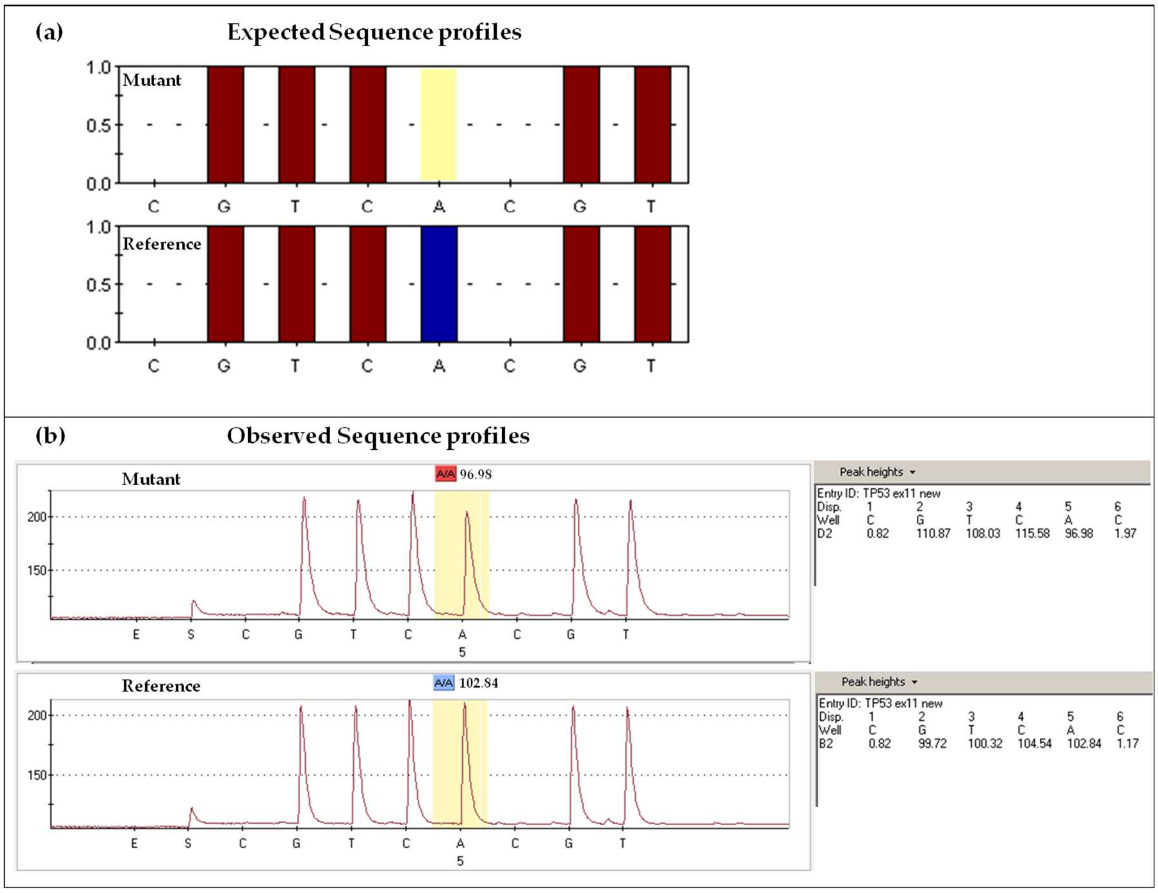

Figure S1. Pyrosequencing of novel somatic mutation *TP53* c.1180delT. (**a**) Expected sequence profiles from pyrosequencing of mutant and reference samples; (**b**) The sequence is in reverse complement. Observed sequence profiles from pyrosequencing. Analyses were performed on genomic DNA from tumor tissue (Mutant) and PBMCs (Reference). The peaks were quantified for mutant sample (A: 96.98/108.03 = 9%) and reference sample (A: 102.84/100.32 = 102%). Results are representative of two independent experiments.

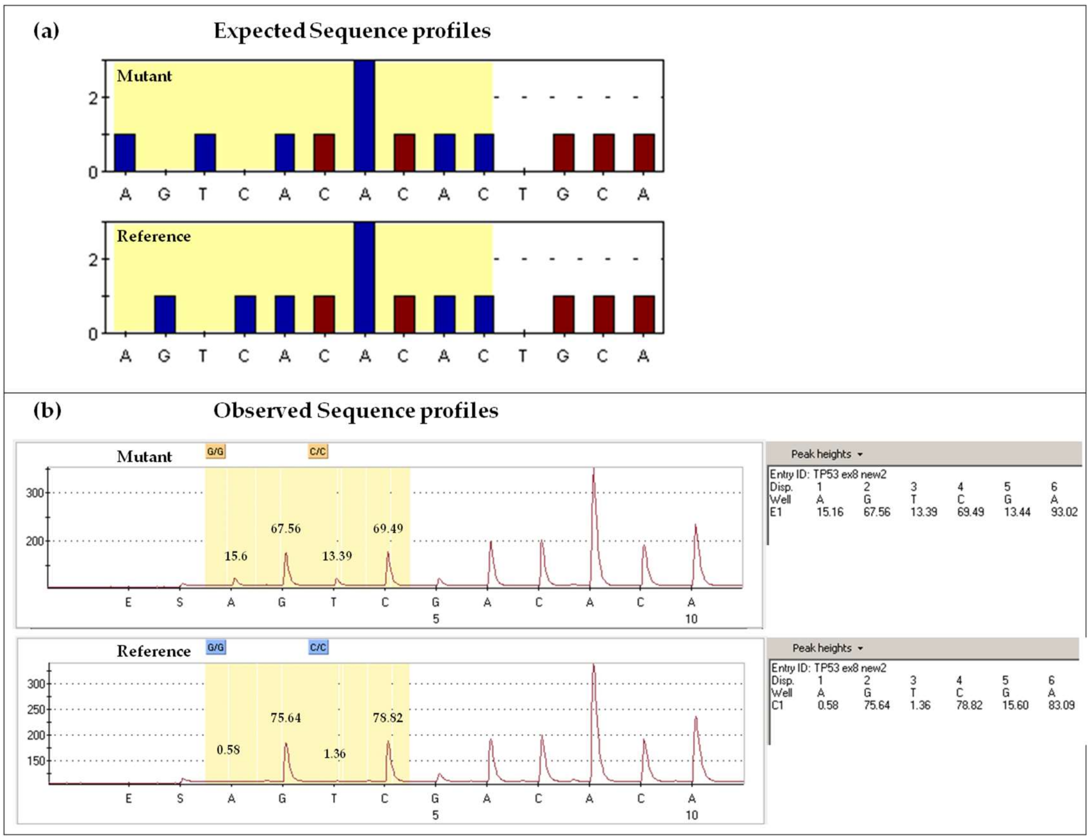

Figure S2. Pyrosequencing of novel somatic mutation *TP53* c.826_827GC>AT. (**a**) Expected sequence profiles from pyrosequencing of mutant and reference samples; (**b**) The sequence is in reverse complement. Observed sequence profiles from pyrosequencing. Analyses were performed on genomic DNA from tumor tissue (Mutant) and PBMCs (Reference). Peaks were quantified for mutant sample (A: 15.16/67.56 = 22%; T: 13.39/69.49 = 19%) and reference sample (A: 0.58/75.64 = 0.8%; T: 1.36/78.82 = 1.7%). Results are representative of two independent experiments.

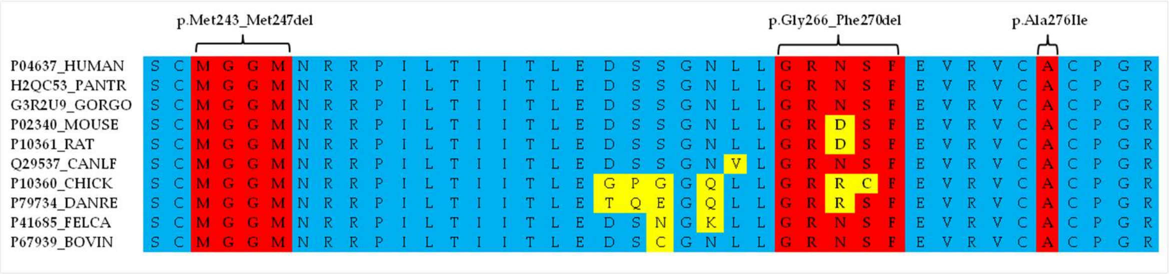

Figure S3. Clustal Omega multiple alignment of the mature p53 protein sequence in different species, including humans in partial exons 7 and 8, highlighting the degree of phylogenetic conservation for the novel *TP53* somatic mutations p53 p.Met243_Met247del (Case ID 305), p.Gly266_Phe270del (Case ID 519), and p.Ala276Ile (Case ID 627). AA residues completely conserved in the positions affected by the novel mutations are colored in red; residues that are not conserved (AA change) are in yellow; residues completely conserved outside the positions affected by the novel mutations are in blue. AA residues in positions affected by the novel mutations p53 p.Met243_Met247del and p53 p.Ala276Ile are strictly conserved in 100% of sequences from different species. Met: methionine; Gly: glycine; Phe: phenylalanine; Ala: alanine; Ile: isoleucine; del: deletion. HUMAN: *Homo Sapiens*; PANTR: *Pan troglodytes*; GORGO: *Gorilla gorilla gorilla*; MOUSE: *Mus musculus*; RAT: *Rattus norvegicus*; CANLF: *Canis lupus familiaris*; CHICK: *Gallus gallus*; DANRE: *Dario rerio*; FELCA: *Felis catus*; BOVIN: *Bos Taurus*. UniProtKB (http://uniprot.org) protein codes are given for each organism.

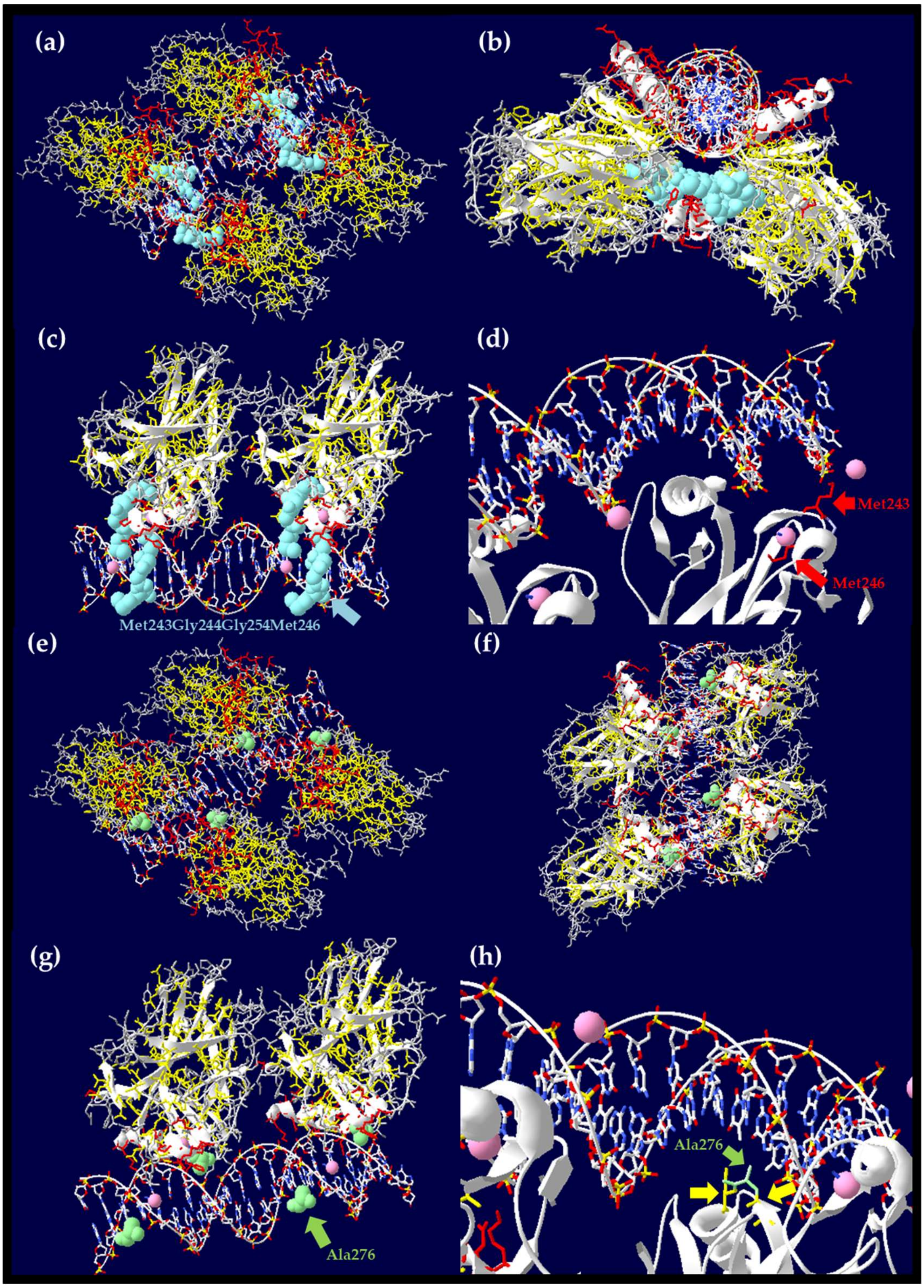

Figure S4. Three-dimensional (3D) structural representation of the p53 core domain highlighting the residues affected by the novel *TP53* somatic mutations found in Case ID 305 (p53 p.Met243_Met247del) and Case ID 627 (p53 p.Ala276Ile), which cluster in highly conserved regions in the DBD. (**a**,**b**) Case ID 305: 3D structural representation of the tetrameric active form of p53 in complex with DNA. Conserved residues Met243, Gly244, Gly245, and Met246 form part of the DNA minor groove binding surface. (**a**) Colored AAs represent residues involved in secondary structures, β-sheet (yellow) and α-helix (red). Met243-Met246 residues are shown in volume structure (blue color); (**b**) Secondary structures are represented in white. AA residues in β-sheet and α-helix motifs are highlighted in yellow and red, respectively; (**c**,**d**) 3D structural representation highlights in detail the position of Met243-Met246 residues in the DNA minor groove binding surface in the DBD

of active p53 (only two isoforms are represented). (**c**) Met243-Met246 (blue volumes) are in proximity to the minor binding surface of DNA. Zinc ions are evident (rose spheres); (**d**) p.Met243_Met247del localizes in the L3 loop (Met237-Pro250), which forms part of the DNA minor groove binding surface. Two flexible residues, Gly244 and Gly245 (purple), flank the Met243 and Met246 residues (red). Zinc ions are evident (rose spheres); (**e**,**f**) Case ID 627: 3D structural representation of the tetrameric active form of p53 in complex with DNA. Conserved residue Ala276 is close to the DNA major groove binding surface in the DBD. (**e**) Colored AAs represent residues involved in secondary structures, β-sheet (yellow) and α-helix (red). Ala276 residues are shown in volume structures (green color); (**f**) Secondary structures are represented in white. AA residues in β-sheet and α-helix motifs are highlighted in yellow and red, respectively; (**g**,**h**) 3D structural representation highlights in detail the position of aliphatic Ala276 residue close to the DNA major groove binding surface in the DBD of active p53 (only two isoforms are represented). (**g**) Ala276 (green volumes) is in proximity to the major binding surface of DNA. Zinc ions are evident (rose spheres); (**h**) the mutation p.Ala276Ile localizes in the short loop (Cys275-Cys277) between the S10 β-sheet and H2 α-helix motifs; Cys275 and Cys277 residues flanking Ala276 are evident (yellow), as well as zinc ions (rose spheres). Structures were created using Swiss-PdB Viewer v4.1 (PDB ID: 4HJE).

Table S1. *In silico* predictive analysis of the novel *TP53* somatic mutations.

Case ID	*TP53* Novel Somatic Mutation	Polyphen [a] (Score)	SIFT [b] (Score)	MutationTaster [c] (*P*)	HSF [d]		
					Splice Site Type	Predicted Signal	Interpretation
305	c.728_739delTGGGCGGCATGA			Disease-causing ($P_{correct}$ = 0.9840)	Acceptor Site	New Acceptor Site<br>ESS Site broken<br>New ESE Site	AS Intronic Activation Potential Splicing Alteration<br>ESS Intronic Alteration No impact on splicing<br>ESE Intronic site Creation No impact on splicing
519	c.795_809delGGGACGGAACAGCTT			Disease-causing ($P_{correct}$ = 1)	Acceptor Site	New Acceptor Site<br>Three ESS Sites broken	AS Intronic Activation Potential Splicing Alteration<br>ESS Intronic Alteration No impact on splicing
627	c.826_827delGCinsAT	Probaly damaging (1.000)	Damaging (0)	Disease-causing ($P_{correct}$ = 0.9999)		No impact on splicing	
738	c.1022dupT			Disease-causing ($P_{correct}$ = 1)	Acceptor Site	New ESS Site<br>ESE Site Broken	ESS exonic site Creation Potential Splicing Alteration<br>ESE site exonic Alteration Potential Splicing Alteration
751	c.1180delT			Polymorphism ($P_{correct}$ = 0.9999)		No impact on splicing	
761	c.573dupT			Disease-causing ($P_{correct}$ = 1)	Acceptor Site	ESE Site Broken	ESE site exonic Alteration Potential Splicing Alteration

[a] PolyPhen v2.2.2r398 (http://genetics.bwh.harvard.edu/pph2) was run using the UniProtKB reference code for human p53 P04637. The PolyPhen score represents the probability that a non-synonymous substitution is damaging; values nearer 1 are more confidently predicted to be deleterious; [b] SIFT Blink (http//:http://sift.jcvi.org/www/SIFT_BLink_submit.html) was run using RefSeq ID NP_000537. The SIFT score is the normalized probability that the amino acid change is tolerated; scores nearer 0 are more likely to be deleterious. The qualitative prediction is derived from this score such that substitutions with a score <0.05 are called 'deleterious' and all others are called "tolerated"; [c] The MutationTaster (http://mutationtaster.org) probability value is the probability of the prediction, i.e., a value close to 1 indicates high 'security' of the prediction; [d] The Human Splice Finder (HSF 3.0.2 released on December 4, 2017; http://umd.be/HSF3/index.html) was used to investigate the potential impact of splicing abnormalities caused by *TP53* novel somatic variants at the non-consensus splice sites, including the effect on the exonic splicing enhancer (ESE) and exonic splicing silencer (ESS). HSF provides scores to value the strength of the splicing-relative sequence motif using corresponding weight matrices. AS: acceptor site.

Table S2. Primers used to amplify distinct *TP53* exons.

**Primers for Sanger Sequencing**		**Primers for Pyrosequencing**	
TP53-Exon5-6-Forw	5′ TGTTCACTTGTGCCCTGACT 3′	TP53-Exon8-ForwF1BIOT	5′ CTGGGACGGAACAGCTTTGA 3′
TP53-Exon5-6-Rev	5′ TTAACCCCTCCTCCCAGAGA 3′	TP53-Exon8-RevR1	5′ CTTGCGGAGATTCTCTTCCTCTG 3′
TP53-Exon6-Forw	5′ GCCTCTGATTCCTCACTGAT 3′	TP53-Exon8-SeqS1Rev	5′ GTCTCTCCCAGGACAG 3′
TP53-Exon6-Rev	5′ TTAACCCCTCCTCCCAGAGA 3′	TP53-Exon11-ForwF1BIOT	5′ ATGTTCAAGACAGAAGGGCCTGAC 3′
TP53-Exon7-Forw	5′ CTTGCCACAGGTCTCCCCAA 3′	TP53-Exon11-RevR1	5′ CTATTGCAAGCAAGGGTTCAAAGA 3′
TP53-Exon7-Rev	5′ AGGGGTCAGAGGCAAGCAGA 3′	TP53-Exon11-SeqS1Rev	5′ GGAACAAGAAGTGGAGAAT 3′
TP53-Exon8-Forw	5′ TTCCTTACTGCCTCTTGCTT 3′		
TP53-Exon8-Rev	5′ AGGCATAACTGCACCCTTGG 3′		
TP53-Exon8-9-Forw	5′ TTGGGAGTAGATGGAGCCT 3′		
TP53-Exon8-9-Rev	5′ AGTGTTAGACTGGAAACTTT 3′		
TP53-Exon10-Forw	5′ CAATTGTAACTTGAACCATC 3′		
TP53-Exon10-Rev	5′ GGATGAGAATGGAATCCTAT 3′		
TP53-Exon11-Forw	5′ AGACCCTCTCACTCATGTGA 3′		
TP53-Exon11-Rev	5′ TGACGCACACCTATTGCAAG 3′		
